# Faculty Development- Is Some Better Than None?

**DOI:** 10.15694/mep.2019.000018.1

**Published:** 2019-01-22

**Authors:** Kelsey Anne Crawford, Timothy J Wood, Karl-André Lalonde, Nancy Dudek

**Affiliations:** 1 University of Ottawa

**Keywords:** Assessment, Faculty Development, In-Training Assessment, Workplace Based Assessment, Feedback

## Abstract

This article was migrated. The article was marked as recommended.

**Introduction:** With the advent of competency-based medical education there is an emphasis on formative workplace based assessment. The quality of these assessments is a concern for medical educators and their trainees. Faculty development (FD) strategies to improve assessment quality have resulted in some success. However, few faculty participate, and those who do are likely more motivated to improve, making it difficult to demonstrate a conclusive benefit. To address these weaknesses, we designed a FD initiative to improve the quality of completed in-training evaluation reports (ITERs). All faculty within a division participated. We hypothesized that clinical supervisors would improve their ITER quality based on feedback, regardless of their own motivation to do so, with a simple, point-in-time intervention.

**Methods:** In this three-phase study, two independent raters used the Completed Clinical Evaluation Report Rating (CCERR) to assess the quality of ITERs completed by all faculty in the Division of Orthopedic Surgery at the University of Ottawa. In phase one, ITERs from the previous nine months were evaluated. In phase two, the participants were aware that their ITERs were being evaluated, but they did not receive feedback. In phase three, participants received regular feedback on their performance in the form of their mean CCERR scores. Mean CCERR scores from the different phases of the study were compared.

**Results:** CCERR scores were similar for all three phases (one: 17.56 ± 1.02, two: 17.65 ± 0.96, three: 17.54 ± 0.75, p=0.98).

**Discussion and Conclusions:** There was no evidence in our study that participants’ improved their ITER quality despite being aware that they were being evaluated and/or receiving feedback. Potentially, this was related to a lack of motivation. Alternatively, the intensity and/or frequency of the feedback may have been inadequate to create change. These results raise concerns that some faculty development may not necessarily be better than none.

## Introduction

Medical education training programs, both at the undergraduate and postgraduate level, need to assess the clinical performance of their trainees to ensure that they are competent to move to the next level of training or into independent practice. In-training evaluation (ITE) by physician preceptors is a common component of many training programs’ assessment process. This assessment is recorded on an In-Training Evaluation Report (ITER). ITERs are also referred to as clinical performance reports, performance assessment forms, clinical performance progress reports and end of clinical rotation reports. ITERs follow the typical format of many workplace based assessment (WBA) tools in that they consist of a list of items on a checklist or rating scale and written comments. Unfortunately, ITERs are often poorly completed, particularly in the case of the poorly performing resident ( Cohen *et al.*, 1993; Speer, Soloman and Ainsworth, 1996; Hatala and Norman, 1999). There is also evidence that clinical supervisors lack knowledge regarding what to document on ITERs and that this is in part responsible for their failure to report unsatisfactory clinical performance ( Dudek, Marks and Regehr, 2005).

With the advent of competency-based medical education (CBME) there is a substantial emphasis on WBA. Although ITERs are less likely to be used in a CBME program of assessment given their summative focus, similar concerns exist surrounding other formative WBA tools with a comparable format (checklist or rating scale with space for written comments). For example, the Daily Encounter Card is commonly used in many emergency medicine programs. Unfortunately, it has similar concerns with regards to quality ( Cheung *et al.*, 2016).

Faculty development (FD) is commonly used to address concerns with regards to the teaching and assessment skills of physicians who supervise medical trainees. FD is anticipated to be essential for a successful transition to CBME ( Dath and Iobst, 2010; Royal College of Physicians and Surgeons of Canada 2017).The Advisory Committee on Educational Outcome Assessment ( Swing *et al.*, 2009) has proposed that assessor training is a key component in addressing the problem of quality assessments in residency programs. Clinical supervisors have also indicated that they want FD programs to help them improve their ability to complete evaluation reports ( Dudek *et al.*, 2005). It seems logical that rater training would improve report quality. However, the literature on this is mixed with success (Holmboe, Hawkins and Huot, 2014; Littlefield *et al.*, 2005; Dudek *et al.*, 2012; Dudek, Marks and Dojeiji, 2013) and failures ( Newble, Hoare and Sheldrake, 1980; Cook *et al.*, 2009).

In addition to the controversy regarding the “trainability” of faculty, there are other concerns with a rater training approach. First, there is some concern that positive improvements noted with regards to rater training might be the result of the participants knowing that they are being observed, a type of Hawthorne effect ( Holden, 2001). This concern is raised because many studies of FD strategies lack a suitable control group ( Dudek *et al.*, 2012; Steinert *et al.*, 2006), which raises the question of whether it was the FD intervention or the monitoring that resulted in the observed improvement. Second, there is the constant concern of FD recruitment ( Rubeck and Witzke, 1998). Notoriously, few faculty participate in FD so the impact of FD is not large. Third, there is the “motivation for change” issue. Potentially, those who chose to participate in FD may be more motivated to change; therefore, even if these strategies are proven successful they may not be universally applicable (i.e. to unmotivated faculty).

To address the dual concerns of recruitment and possible Hawthorne effect, Dudek et al. developed a FD program designed to improve completed ITER quality. This program provided similar content to an existing FD workshop that demonstrated improved ITER quality ( Dudek *et al.*, 2012; Dudek, Marks and Dojeiji, 2013), but used an “at-home” format incorporating a feedback component ( Dudek *et al.*, 2013a) and a control group. An improvement in ITER quality was noted in the intervention group, but not in the control group, suggesting that the information was creating a change in ITER quality. As well, this “at home” program had greater participation than the “in-person” workshop. However, recruitment remained an issue and limited the power of the study. In addition, the Dudek et al., study did not address the motivation issue, as participation was voluntary. Therefore, it can be assumed that those who agreed to participate were motivated to improve their ITER quality.

To build on this previous work, the current study was designed to address these two limitations: low participation rate and possible motivational differences. First, we made participation in the study extremely simple as participants were no longer responsible for sending research team their ITERs for assessment. Their ITERs were simply provided to the research team by the program’s administrative assistant. Second, we had all supervisors in one postgraduate residency participate so that we would be offering this FD initiative to everyone and not just those who chose to participate.

The objectives of this study were to determine if clinical supervisors improved their ITER quality when they knew that they were being monitored and whether they could improve their ITER quality based on feedback, regardless of their own motivation to do so. If successful, this practical intervention to improve ITER quality could be applied in any residency program.

## Methods

This study involved assessing the quality of the ITERs completed in the Orthopedic Surgery residency-training program at the University of Ottawa. Two raters using the Completed Clinical Evaluation Report Rating (CCERR) assessed the quality of the completed ITERs. The project was divided into 3 phases: 1) participants were unaware that their ITER quality would be assessed; 2) participants were aware that their ITER quality would be assessed but they did not receive any feedback and 3) participants received feedback in the form of their mean CCERR scores, a copy of the CCERR tool and a copy of the ITERs that they had completed as per the protocol used by Dudek, Marks, Bandiera et al. (2013). In this previous study there were two feedback groups: one group that received the items listed above and a second that included those items plus a feedback guide that provided additional information about how to improve their ITERs. No difference was noted between the groups ( Dudek *et al.*, 2013a). Therefore, given that we wanted the intervention in our study to be a simple as possible for faculty to participate in we chose to exclude the feedback guide for the present study. The mean CCERR scores from each phase were compared.

### Participants

All Orthopedic Surgeons affiliated with The Ottawa Hospital Orthopedic Surgery residency program who completed ITERs between December 2012 and August 2015 were participants in this study. They provided consent at the start of phase two (see description below). This consent included looking at their past ITERs (phase 1 - see below). There was no additional work required outside of their typical educational responsibilities with the exception of reviewing the feedback sent via e-mail in phase three.

### Experimental Design

#### Phase One (December 2012 - August 2013)

Participants were informed of the study and told that their ITERs were being collected and evaluated for the next two years starting in September 2013. They were also informed that phase one involved rating the preceding 9 months of their completed ITERs. Nine months was chosen, as participants would typically have completed 2 to 3 ITERs during that time period. This served as a baseline assessment of participants’ ITER quality when they did not think that their ITER quality was being assessed (a control phase).

#### Phase Two (September 2013 - November 2014)

During this phase, participants were aware that their ITERs were being studied, but they did not receive their CCERR scores. This phase addressed whether simply being aware that they were in a study affected participant performance. To help minimize bias, ITERs from phases one and two were rated at the same time and raters were blinded to phase.

#### Phase Three (December 2014 - August 2015)

Participants received feedback on their ITER quality in the form of the mean CCERR score for each item on the CCERR and the total CCERR score for each ITER that they completed. This was provided to them on an actual CCERR tool so that they were informed of what each item was assessing. They also received a copy of the corresponding ITER, for reference. This protocol, which was developed for the previous study ( Dudek *et al.*, 2013a), was designed based on commentary provided by past workshop participants that the CCERR is so self explanatory that after just reviewing it along with their own ITERs it was easy to know how to improve their ITER quality ( Dudek *et al.*, 2013). Phase three data collection started after they had received their first feedback in December 2014. The first set of feedback included CCERR scores from ITERs completed from July to November 2014. Note that the CCERR results from July to November 2014 were included in phase two (as they had not yet received this feedback). This phase addressed whether participants improve their performance based on feedback, regardless of their motivation to do so.

Given that the CCERR score was to be relayed back to the participant in time for them to complete further ITERs, the raters were not blinded to study phase during this time. This phase was intended to evaluate the impact of feedback on their performance.

### ITERs

ITERs were printed in paper form and de-identified (both preceptor and resident) by an administrative assistant before they were given to the raters for review.

### CCERR

Results from the Completed Clinical Evaluation Report Rating (CCERR) have demonstrated evidence for validity ( Dudek *et al.*, 2008; Dudek *et al*., 2012; Dudek *et al.*, 2013a). It is used to rate the quality of ITERs completed by physician supervisors regardless of the style of the form provided, it includes a list of items to be evaluated on a checklist or rating scale and a space for comments ( Dudek *et al.*, 2008).Nine items are rated on a five point scales (where a three is defined as acceptable) resulting in a total score that ranges from nine to forty-five. For reference, a total CCERR score of ~16 corresponded with ITERs rated as poor quality by experts, a score of ~24 corresponded to average quality, and a score of ~32 was considered high quality ( Dudek *et al.*, 2008).

### Raters

Two physician raters scored each ITER using the CCERR. Previous research demonstrated that two physician raters per ITER are sufficient for adequate reliability ( Dudek *et al.*, 2008). Physician raters can reliably use the CCERR without the need for additional rater training beyond following the written instructions on the CCERR ( Dudek *et al.*, 2008). Physician raters were blinded to the staff and resident associated with each ITER.

Phase three of our study required that the ITERs be evaluated in a timely fashion so that participants could receive feedback continually. Given that one of our objectives was to create an intervention that could be replicated in any residency program, the physician rater in this scenario would be aware that this intervention was occurring. This raises a concern for bias, as raters would know that the supervisors were getting feedback and may be expecting improvement. To fully re-create this scenario, and then evaluate for bias, we had the two physician raters be two of our co-investigators (who were aware of the intervention phase). At the conclusion of the study, all ITERs were rated by two additional independent raters who were blinded to participant, resident **and** phase to determine if there were differences between the blinded-to-phase and unblinded-to-phase ratings.

### Analysis

Two physicians rated each ITER using the CCERR. A total CCERR score was determined for each submitted ITER by summing the ratings on each item of the CCERR assigned by a specific rater. For each participant in a phase, a mean total CCERR score was determined by averaging across the forms a participant submitted. These total CCERR scores for each participant were analyzed with phase (phase one, phase two, phase three) as a repeated measures factor. Correlations between raters for each phase were also determined.

To explore the potential effect of rater bias, all ITERs were evaluated by two separate independent physician raters who were be blinded to study phase, as well as to resident and physician, at the completion of the study. The analysis above was repeated. In addition, the CCERR ratings from the blinded raters were compared to the original CCERR scores using rater type (blind, unblind) as a repeated measures factor and correlations

## Results/Analysis

There were nineteen members of the Division of Orthopedic Surgery who were involved in resident ITER completion at the initiation of the study. This included co-investigator KL; however, he was not eligible to participate, as he was a rater in the study. Two surgeons had only recently joined the division and were deemed ineligible because they did not have phase one ITERS available. All sixteen remaining members of the Division of Orthopedic Surgery at The Ottawa Hospital agreed to participate, resulting in a 100% participation rate for eligible participants. However, three participants did not submit ITERs in all phases of the study and they were therefore not included in the study analysis.

### ITER Quality

The thirteen participants completed one hundred and seventy one ITERs in total. Forty-seven ITERs were submitted in phase one, forty-five ITERs in phase two, and seventy-nine ITERs in phase three. Table 1 illustrates the number of ITERs submitted by each participant and their mean total CCERR score for each phase.

**Table 1.** Participant mean total CCERR scores by phase

Participant	Number of Phase 1 ITERs	Mean CCERR score (± standard error) Phase 1	Number of Phase 2 ITERs	Mean CCERR score (± standard error) Phase 2	Number of Phase 3 ITERs	Mean CCERR score (± standard error) Phase 3
1	5	23.80	3	26.17	5	24.20
2	3	18.50	8	13.13	4	17.13
3	3	17.67	4	17.75	2	16.75
4	3	16.67	7	16.14	3	20.83
5	3	22.17	6	22.50	11	18.81
6	1	17.50	2	17.25	1	17.00
7	4	9.63	7	14.71	5	15.70
8	4	20.13	6	18.75	2	19.00
9	3	12.83	5	14.10	1	17.00
10	6	15.08	7	17.64	4	13.88
11	3	17.67	3	18.00	3	14.83
12	4	17.75	7	17.07	2	17.00
13	3	18.83	9	16.28	4	15.86
Total (of eligible participants, n=13)	47	17.56 ± 1.02	45	17.65 ± 0.96	79	17.54 ± 0.75

Note that the total CCERR score has a range of 9 to 45.

### Comparative Data

The mean CCERR score for phase one was 17.56 ± 1.02, phase two was 17.65 ± 0.96 and phase 3 was 17.54 ± 0.75. There was no significant improvement in CCERR scores (F(2,24) = .012, p=.98, η ^2^_p_= .001). However, there was a significant interaction between rater and phase (F(2,24) = 5.013, p=.02, partial eta = .295).This interaction appears to be as a result of a difference in phase three, as compared to phase one and two. Raters were highly correlated for phases one and two (r(11) = 0.95, p<.001 and r(11)= 0.97, p<.001, respectively). In phase three there was a moderately high correlation of r(11)=0.68, p=.01. The raters were not blinded during this time, so the lower correlation raises the question of rater bias in phase three.

To address the issue of blinding, two independent blinded raters with previous experience using the CCERR rated all ITERs at once. The mean CCERR scores were 14.21 ± 0.71 (phase one), 15.11 ± 0.60 (phase two) and 14.46 ± 0.64 (phase three). The blinded raters also showed no difference in mean CCERR score between the phases. F(2,24)=1.23, p=0.31, η ^2^_p_= 0.09. Unlike the unblinded raters, there was no interaction between rater and phase (F(2,24) =.532, p=.59, η ^2^_p_= .04).

The CCERR scores from the blinded raters were lower than from the unblinded raters. F(1,12) = 114.08, p< 0.001, η ^2^_p_= 0.91. However, despite the fact that they rated the ITERs lower on the CCERR than the unblinded raters, they were rating them consistently as evidenced by the strong correlations by the two groups of raters ( phase 1: r(11) = 0.94, p<.001, phase 2: r(11) = 0.93, p<.001 and phase 3: r(11) = 0.93, p<0.001).

Overall, there was no evidence of improvement from participants’ knowing they were being monitored (no improvement phase one versus phase two) or from the feedback provided (phase one or two versus phase three).

## Discussion

The purpose of this study was to determine if a practical, low-intensity intervention provided to an entire division of clinical supervisors, with potentially differing motivations to improve, would improve ITER quality. Quite simply, it did not demonstrate any effect. No significant improvement was found from participants knowing they were being monitored or from receiving feedback in the form of their CCERR score.

Why didn’t this intervention work when studies with similar content have been successful ( Dudek *et al*., 2012; Dudek *et al.*, 2013a)? Is it possible that this group was simply not as good at completing quality ITERs and, therefore, not able to use the feedback like previously studied groups? The results do not support this possible explanation. The mean CCERR scores were in the poor to average quality range (poor ~16 and average ~24) ( Dudek *et al.*, 2008). However, this level of performance is consistent with the quality of ITERs completed by participants in all previous studies (ie. poor to average range) where an improvement was noted following the faculty development intervention ( Dudek *et al.*, 2008; Dudek *et al.*, 2012; Dudek, Marks and Dojeiji 2013; Dudek *et al.*, 2013a).

A more likely possibility is that the feedback was not timely enough. It is well known that in order for feedback to be useful it must be provided in a timely fashion ( Hunt, 1992). There must also be an opportunity to use the feedback soon after receiving it ( Doran, 1981). Our feedback was provided at three time points over a nine-month period; however, not all participants received three chunks of feedback, as ITER submission was variable. The variability may have occurred because they did not have a resident for a part of the year or the ITER was not completed in a timely fashion. In fact, some participants clumped the completion of all their ITERs for that time period together on the same day and therefore would not have had an opportunity to improve. In other situations, they may have received the feedback reasonably soon after they completed their ITER but did not have the opportunity to complete the ITER for several months and hence forgot some of the observations that may have been made on how they could improve.

There is also a chance that the amount of feedback was not adequate. First, we are assuming that the participants actually read their e-mail and reviewed their results and this may not be true. Even if they did review the CCERR, their CCERR scores and their ITERs, it is possible that it was simply not enough information to create change. A previous study demonstrated improvement in ITER quality with participants receiving the exactly the same information, so, we had anticipated similar results with this study ( Dudek *et al.*, 2013a). However, in the previous study the intervention group was mixed with some participants receiving only the above information and others receiving that information plus a generic feedback guide on how to improve ITER quality. No difference was noted between these groups. Therefore, in this study we did not include the feedback guide, as we wanted to keep things simple for the participants. It is practically important to determine the minimally effective dose of feedback since time is often cited as a reason for individuals to not participate in FD and is therefore a potential barrier when trying to deliver FD to the masses. It is notable that the previous study may have been underpowered to detect a difference between the groups. If a difference does exist it is possible that we may have been too “streamlined” in the amount of information provided.

Participants’ underlying motivation to improve may also have had an impact on the results. It is known that clinical supervisors’ underlying motivation can affect their teaching quality ( Cate, Kusurkar and Williams, 2011), so why not their ITER quality? Participants seven and nine improved during phase three. Although our study was not powered to determine statistical significance at the individual level, it is possible that these differences may be related to underlying participant motivation (ie. those who improved were motivated to do so). Improving ITER quality may not have been seen as a priority for the majority of our participants amongst the many pressures in a residency program. In addition, faculty are aware that there is currently a shift toward CBME in Canadian residency programs. They may believe that other forms of assessment will ultimately replace ITERs, leading them to be less interested and motivated to change.

The self-determination theory ( Deci and Ryan, 2000) proposes that a person’s behavior is determined not only be the level of motivation, but also by the type of motivation, intrinsic or extrinsic. Intrinsic motivation is when an individual pursues an activity out of personal interest and extrinsic motivation is when the activity is pursued in order to obtain a reward or avoid loss or punishment ( Deci and Ryan, 2000). In this study, we did not try and specifically increase extrinsic motivation (ie. no external reward for improvement or punishment for lack of improvement) and therefore relied on participants’ baseline intrinsic motivation. It is possible that if the results were not anonymous (ie. the program director or director of assessments was aware of the individuals performance), or that there was an external incentive or punishment related to performance, that the participants’ would be more motivated to improve, potentially leading to improved results. We attempted to answer some of these questions through a post study email survey. However, we did not receive any responses.

It is relevant to note that our blinded raters were more stringent and provided lower CCERR scores than the unblinded raters; however, the numerical value of this difference was not large (ie. 2-3 points on the scale). Despite being lower, their scores were highly correlated, suggesting that they rated the ITERs consistently regardless of blinding. For formative faculty development purposes unblinded raters should be adequate.

Ultimately, there was no additional work required by the participants to participate in this intervention other than looking at their CCERR scores. The low-commitment nature of the intervention potentially did appear to overcome the issue of recruitment, a commonly cited barrier to FD participation ( Rubeck and Witzke, 1998), as everyone agreed to participate.

Given the shift in medical education towards competency based medical education curricula there is a substantial need for faculty development ( Dath and Iobst, 2010). As this shift involves all clinical faculty there has been a push for short bursts of point-in-time faculty development ( Royal College of Physicians and Surgeons of Canada 2017). Although the ITER will likely not be a dominant part of trainee assessment, CBME curricula relies heavily on workplace-based assessment tools that follow a very similar format to ITERs (items on a checklist and narrative comments) which are known to suffer from some of the same quality concerns as ITERs ( Royal College of Physicians and Surgeons of Canada 2017). As a result faculty development on assessment quality will be key. Strategies to access all clinical supervisors, rather than only those who are already interested in improving and volunteering for FD, are needed ( Steinert *et al.*, 2006). Decreasing the time commitment required to participate seems logical; however, there is likely a minimal required intensity of faculty development in order to observe positive change.

## Conclusion

Overall, the results of our study raise the concern that short bursts of feedback directed at clinical teachers on relevant activities is not necessarily going to be successful. It is imperative that medical educators stop and think as we move forward with faculty development initiatives for our new CBME curricula. It is undoubtedly going to be a challenge to capture all clinical teachers and have them improve their assessment quality. As this study clearly demonstrates, it is not going to be so simple as some faculty development is not necessarily better than no faculty development. Future studies will need to determine the optimal, effective approaches to faculty development that are practical to employ with all of our clinical teachers.

## Take Home Messages

• Faculty development (FD) strategies to improve assessment quality have resulted in some success but questions remain regarding the necessary motivation and time commitment by faculty to see these results.
• A simple, point-in time feedback intervention was given to all faculty in one division.
• Faculty did not improve their In Training Evaluation Report (ITER) quality despite knowing they were being monitored and despite receiving feedback.
• These results suggest that some faculty development may not necessarily be better than none.
• Medical educators should be cautious in their expectations regarding the impact of small FD strategies to a general population of faculty.

## Notes On Contributors

**Dr. Crawford** is a Lecturer, Faculty of Medicine, University of Ottawa, Ottawa, Ontario, Canada. Orcid ID https://orcid.org/0000-0001-7794-4358

**Dr. Dudek** is a Professor, Faculty of Medicine, University of Ottawa, Ottawa, Ontario, Canada. Orcid ID https://orcid.org/0000-0002-6178-406X

**Dr. Wood** is a Professor, Department of Innovation in Medical Education (DIME), University of Ottawa, Ontario, Canada. Orcid ID https://orcid.org/0000-0001-9177-704X

**Dr. Lalonde** is an Assistant Professor, Department of Surgery, University of Ottawa, Ottawa, Ontario, Canada. Orcid ID https://orcid.org/0000-0002-1634-6474

## Declarations

The author has declared that there are no conflicts of interest.

## Ethics Statement

The Ottawa Health Science Network Research Ethics Board approved this project (20130300-01H).

## External Funding

Funding support for this study was obtained from the Department of Innovation and Medical Education (DIME), University of Ottawa, Education Research Grant.
