# Supplementary material for: Faculty Development- Is Some Better Than None?
Source: MedEdPublish (2016). 2019 Jan 22;8:18. [Version 1] doi: 10.15694/mep.2019.000018.1 (PMC10712536; doi:10.15694/mep.2019.000018.1)
Supplement: Peer review history

# Reviewer report: Reviewer response for version 1

**DOI:** 10.21956/mep.19753.r29639

**Published:** 2019-04-29

**Authors:** Saee Deshpande

**Affiliations:** 1 Maharashtra University of Health Sciences, Nashik- VSPM Dental College Nagpur

This review has been migrated. The reviewer awarded 5 stars out of 5 A very interesting and brave paper. I often experience this at our institution, faculty do not take up assessment and for that matter teaching seriously or more student centric so to say. Its disheartening in the beginning but I think its an iterative process. We should try and investigate Why's of the situation..we cant always blame on lack of intrinsic motivation, other factors such as institutional environment, peer characteristics matter as well.A well written paper, please follow up with faculty for qualitative analysis..

Reviewer Expertise:

NA

No decision status is available

# Reviewer report: Reviewer response for version 1

**DOI:** 10.21956/mep.19753.r29640

**Published:** 2019-03-12

**Authors:** Susan Van Schalkwyk

**Affiliations:** 1 Stellenbosch University

This review has been migrated. The reviewer awarded 4 stars out of 5 As faculty development continues to find traction in the context of health professions education, studies such as the one described in this article are important. It is incumbent on those who plan, design and facilitate faculty development initiatives to be critically reflective on their work, not only because it is important to know that what they are doing is indeed ‘better’, but also because often those who engage in these initiatives do so in the context of multiple other competing demands on their time and attention. The authors are to be commended for demonstrating such criticality and for reporting ‘negative’ results – in so doing reminding us that faculty development activities are in and of themselves not necessarily always ‘good’. I would agree with the other reviewers who call for further, possibly qualitative, work in this area although it does appear that the study reported on builds on a series of early investigations. I found the framing of the intervention as ‘faculty development’ most interesting, given that its approach seemed to be largely based on the provision of feedback on practice. We are reminded of the many different ways in which our practice as health professions educators can be strengthened.

Reviewer Expertise:

NA

No decision status is available

# Reviewer report: Reviewer response for version 1

**DOI:** 10.21956/mep.19753.r29638

**Published:** 2019-01-31

**Authors:** Leila Niemi-Murola

**Affiliations:** 1 University of Helsinki

This review has been migrated. The reviewer awarded 4 stars out of 5 Thank you for iviting me to review this interesting article. The quality of workplace-based assessment is an issue indeed. In my country there are not many tutors who have some kind of pedagogical education and we do not have a tradition of giving formative feedback, either. Thus, assessment of clinical performance evokes anxiety in the clinical educators.The authors were concerned about incomplete recordings, especially when poorly performing residents were concerned. I am not familiar with the Canadian system, and I wonder if the authors had seen the actual clinical encounters or did they just evaluate the quality of the given ITER reports. Were the clinical encounters standardized or live encounters in real clinical settings? Surprisingly, the intervention was not successful and the authors discuss about the effect of underlying motivation of the participants. They gave their informed consent from the phase II onwards, but they did not volunteer to the study. The authors discuss about types of motivation – perhaps the participants did not have enough intrinsic motivation to improve. This is a great effort for faculty improvement, but it is an enormous task and I do hope that the authors keep on trying.

Reviewer Expertise:

NA

No decision status is available

# Reviewer report: Reviewer response for version 1

**DOI:** 10.21956/mep.19753.r29641

**Published:** 2019-01-27

**Authors:** Megan Anakin

**Affiliations:** 1 University of Otago

This review has been migrated. The reviewer awarded 4 stars out of 5 Thank you for inviting me to review this article. Like Trevor Gibbs, I found article thought-provoking. I am wondering if the authors might consider adopting a different theoretical approach to analyse the data in a future study of their intervention. I’m thinking of the work by Patricia O’Sullivan and David Irby (see: O'sullivan PS, Irby DM. Reframing research on faculty development. Acad Med. 2011. 86(4):421-8.) Perhaps taking a view about feedback in a system of assessment might also be helpful. The work of work of Liz Malloy and David Boud might provide another starting point for thinking (For example see: Boud D. Feedback: Ensuring that it leads to enhanced learning. Clin Teach. 2015. 12(1), 3-7. Boud D. Molloy E. (Eds.). Feedback in higher and professional education: understanding it and doing it well. Routledge. 2013.). I look forward to reading about the next phase of the study.

Reviewer Expertise:

NA

No decision status is available

# Reviewer report: Reviewer response for version 1

**DOI:** 10.21956/mep.19753.r29642

**Published:** 2019-01-22

**Authors:** Trevor Gibbs

**Affiliations:** 1 AMEE

This review has been migrated. The reviewer awarded 4 stars out of 5 Thank you for asking me to review this very interesting paper; interesting because it is brave enough to announce negative results but it creates more questions than answers.I was a little confused at first with the authors' use of the terms assessment and evaluation- this often causes some confusion. However this was not enough to detract from the paper. We can often be blamed for assuming that faculty development is the answer for all things, it solves a major problem and by including modern methods of assessment with feedback indices a feel-good factor. This paper shows that complacency is these areas is something that we cannot afford. I feel we fail to consider internal motivation and other factors that affect our enthusiasm to facilitate learning in our students, we expect every faculty member to be interested and passionate about teaching and put quantity before quality.I would encourage the authors to follow up this interesting paper with a more quality-based one that attempts to uncover the "feelings" of these faculty.A very important paper for all curriculum developers to read

Reviewer Expertise:

NA

No decision status is available
